# Depicting the Profile of METTL3-Mediated lncRNA m6A Modification Variants and Identified SNHG7 as a Prognostic Indicator of MNNG-Induced Gastric Cancer

**DOI:** 10.3390/toxics11110944

**Published:** 2023-11-20

**Authors:** Tong Liu, Yanlu Feng, Sheng Yang, Yiling Ge, Tianyi Zhang, Jie Li, Chengyun Li, Ye Ruan, Bin Luo, Geyu Liang

**Affiliations:** 1 Institute of Occupational Health and Environmental Health, School of Public Health, Lanzhou University, Lanzhou 730000, China; liutongseu@163.com (T.L.); lichengyun@lzu.edu.cn (C.L.); ruany@lzu.edu.cn (Y.R.); luob@lzu.edu.cn (B.L.); 2 Key Laboratory of Environmental Medicine Engineering, Ministry of Education, School of Public Health, Southeast University, Nanjing 210009, China; 220203810@seu.edu.cn (Y.F.); 101300318@seu.edu.cn (S.Y.); geyiling11@163.com (Y.G.); tyi_zhang@163.com (T.Z.); lijies7@163.com (J.L.)

**Keywords:** MNNG, m6A RNA methylation, gastric cancer, METTL3, SNHG7

## Abstract

As a representative example of an environmental chemical carcinogen, MNNG exposure is closely associated with the onset of gastric cancer (GC) where N6-methyladenosine (m6A) RNA methylation tends to be the critical epigenetic event. However, the effect of m6A modification on long non-coding RNAs (lncRNAs) in MNNG-induced GC onset is still unclear. To address the above issue, based on the Methylated RNA immunoprecipitation sequencing (MeRIP-seq) data of MNNG-induced malignant cells (MCs) and GC cells, we comprehensively analyzed the MNNG exposure-associated vital lncRNAs. MeRIP-seq analysis identified 1432 lncRNA transcripts in the MC cell, and 3520 lncRNA transcripts were found to be m6A modified in the GC cell, respectively. Gene ontology (GO) and Kyoto Encyclopedia of Genes and Genomes (KEGG) analysis revealed that MNNG exposure could spark cellular localization change, which might be the critical cellular note variation for malignant transformation. We demonstrated that METTL3 is responsible for N6 methylation of lncRNAs and identified SNHG7 as a downstream target of METTL3. More importantly, we observed that SNHG7 was progressively up-regulated during gastric carcinogenesis by MNNG exposure. Finally, we investigated SNHG7 expression in different stages of GC malignancies and found that elevated SNHG7 expression correlated with advanced clinical features and poor prognosis in GC. In conclusion, our study found for the first time that METTL3 regulates the m6A methylation level of lncRNA SNHG7 and its expression in MNNG exposure-induced GC, suggesting that SNHG7 as a predictive biomarker or therapeutic target for GC.

## 1. Introduction

Gastric cancer (GC) is reported to be the fourth most common neoplasm and the third leading cause of cancer-related deaths globally in 2020 [1]. Despite current improvements in diagnostic and therapeutic strategies, the 5-year survival rate for GC remains high, resulting in a poor prognosis for most patients [2]. Unfortunately, the onset of GC is trending younger and becoming more malignant and more drug-resistant [3]. As one of the representative substances of environmental chemical carcinogens, 1-Methyl-3-nitroso-1-nitroguanidine (MNNG) has been closely associated with the onset of gastric cancer (GC) [4] while the underlying mechanisms remain largely unknown. Therefore, there is an urgent need to identify novel biomarkers and therapeutic targets for the diagnosis and treatment of GC.

Recent studies have highlighted the involvement of N6-methyladenosine (m6A) methylation in the pathogenesis and development of GC [5], in which long non-coding RNAs (lncRNAs) play an important role [6,7]. Methyltransferase-like 3 (METTL3) is a vital component of the m6A methyltransferase complex (MTC) [8] and increasing evidence suggests that METTL3 plays an essential role in GC initiation and progression through m6A modification [5,9]. Studies have shown that m6A modifications can act as structural “switches” to facilitate the binding of lncRNAs to RNA-binding proteins [10], further contributing to the involvement of lncRNAs in cell proliferation, metastasis, and invasion [11,12]. Therefore, further identification of m6A-related lncRNAs in GC tumorigenesis is imperative.

In the present study based on methylated RNA immunoprecipitation sequencing (MeRIP-seq) analysis, we screened out a penal of MNNG exposure-associated vital lncRNAs. Then, key lncRNA expression profiles were further verified by sequencing data from public databases and by using qRT-PCRs to detect the expression of lncRNAs in different cells. Finally, downstream target lncRNA SNHG7s regulated by METTL3 were identified by combining correlation analysis, interaction analysis, m6A site prediction, and prognostic survival analysis.

## 2. Materials and Methods

### 2.1. Public GC Data Analysis

RNA sequencing data from The Cancer Genome Atlas (TCGA; http://www.cbioportal.org, accessed on 12 June 2023) dataset were extracted using R 3.5.1 (https://cran.r-project.org/ accessed on 12 June 2023) (level 3). The exclusion criteria were as follows: (1) cases with a pathological diagnosis other than stomach adenocarcinoma (STAD); (2) STAD patients with concomitant malignancies; and (3) patients with incomplete data. A total of 415 STAD cases and 32 normal controls were included in the analysis until 17 October 2017, in accordance with the NIH guidelines and the TCGA Data Access Policy. The patients were categorized based on the 8th American Joint Committee on Cancer (AJCC) TNM staging system [13]. DNA methylation and the clinical features in TCGA GC cohorts were analyzed using the UALCAN tool [14].

The correlation between SNHG7 expression and the survival status of GC patients was explored using the Kaplan–Meier plotter [15], which analyzed data from the Gene Expression Omnibus (GEO) dataset consisting of 875 individuals with survival outcome information. The median overall survival (OS) time was 28.9 months, and the median progression-free survival (PFS) was 18.3 months. Kaplan–Meier survival curves (K m curve) and log-rank tests were performed using IBM SPSS Statistics 23.0 (SPSS Inc., Chicago, IL, USA) to assess survival data. The diagnostic value of METTL3 was evaluated using the receiver operating characteristic (ROC) curve.

### 2.2. Clinical Specimens and Data

GC tissues and paired adjacent normal tissues (*n* = 40) were obtained from individuals who underwent surgery at Zhongda Hospital, which is affiliated with Southeast University. Bio-samples were later stored in RNA (Ambion, Austin, TX, USA) and frozen in a low-temperature freezer at −80 °C. The study was approved by the Ethics Committee of Zhongda Hospital, affiliated with Southeast University, and written informed consent was obtained from each participant. The clinical features of GC patients are detailed in Supplementary Table S1.

### 2.3. Cell Lines and Cell Culture Transfection

The human gastric epithelial cell line (GES-1) and gastric cancer cell line (HGC-27, AGS) were obtained from the Guangzhou Cellcook Biotech Co., Ltd. (Guangzhou, China) Cell lines were cultured in a humidified incubator with 5% CO_2_ at 37 °C in RPMI-1640 medium (GES-1; Gibico, NY, USA) or medium MEM (HGC-27; Thermo et al., Waltham, MA, USA) supplemented with 10% fetal calf serum (FBS), 100 μg/mL penicillin, and 100 μg/mL streptomycin. According to our previous study, the synthetic METTL3 knockdown plasmid and the corresponding negative control were transfected into cells using Lipofectamine 3000 (Invitrogen).

### 2.4. MNNG-Induced Malignant Transformation of GES-1 Cell

The malignantly transformed cells derived from GES-1 (MC cells) were treated with long-term exposure to N-methyl-N-nitro-N’-nitrosoguanidine (MNNG, Sigma-Aldrich, St. Louis, MO, USA). The MNNG exposure condensate was calculated and dissolved in dimethylsulfoxide (DMSO) to give a stock concentration of 1.0 mmol/L.

GES-1 were treated with MNNG at a concentration of 5 × 10^−5^ mol/L for 24 h in the dark for each passage. After MNNG exposure, the cells were cultured in a standard RPMI-1640 medium, which was changed every 48 h. This exposure process continued for 20 weeks, with the cells going through 40 passages (MC-40) until the malignant phenotype was achieved.

### 2.5. Quantitative Real-Time PCR (qRT-PCR) and Plasmid Transfection

RNA extraction from the GC and MC cells and quantitative real-time PCR (qRT-PCR) procedures were conducted as previously described [16]. RNA was converted to cDNA by a two-step reverse transcription process, followed by a real-time PCR using a the StepOneplus real-time PCR system (Applied Biosystems, Foster City, CA, USA) to detect the expression of the target gene.

All mRNA and lncRNA primers were purchased from General Biotech Co., Ltd. (Shanghai, China). The primer sequences for mRNA and lncRNA and housekeeping genes are listed in Table 1. The comparative Ct method was used for the fold change, and the data were analyzed using the relative 2^−ΔΔCt^ method. 

The shMETTL3 (short hairpin RNA METTL3) and negative control genes were synthesized by Hanbio Biotechnology Co., Ltd. (Wuhan, Hubei, China). Confirmed METTL3 shRNA targeting sequences and the reference gene are listed in Supplementary Table S2. The transfections of plasmids into cells were conducted by the Lipofectamine 2000 kit (Invitrogen, Carlsbad, CA, USA). The infection efficiency was confirmed via qRT-PCR and western blot analysis presented in Supplementary Figure S1.

### 2.6. Western Blotting Analysis

According to the protein concentration measured by BCA, add ultrapure water and 5× SDS loading buffer. The protein samples from cells were separated through 10% SDS-PAGE and then transferred onto PVDF membranes (Millipore, Darmstadt, Germany). Then, blocking with 5% milk in TBST for 2 h, the membranes were incubated with primary antibodies (METTL3 ab195352, 1:1000; GAPDH ab181602) overnight at 4 °C.

### 2.7. RNA Extraction and qRT-PCR

Total RNAs were extracted from the transfected ShMETTL3 and paired with negative control of HGC-27 and MC-40 cells. MeRIP assays were performed using the Magna MeRIPTM m6A Kit (Millipore) according to the manufacturer’s instructions. Poly(A) RNA was purified from 50 μg total RNA using Dynabeads Oligo (dT) and then fragmented into small pieces using the Magnesium RNA Fragmentation Module (NEB, cat. e6150, Ipswich, MA, USA).

### 2.8. Scratch Wound Healing Assays

The cells were seeded in a six-well plate at a density of 5 × 10^5^ cells per well, incubated until they reached 90% confluence, and scratches were made on the cell monolayer using a sterile 200 μL pipette tip. The cells were then incubated for an additional 48 h in a 37 °C incubator with 5% CO_2_. The migration distance of the cells was captured using the FSX100 Bio-Image System (Media Cybernetics, Rockville, MD, USA). The percentage of wound closure, indicating the extent of cell migration, was calculated and evaluated using the ImageJ software version 6.1.

### 2.9. Cell Invasion Assays

After 24 h of incubation, cells at a density of 1 × 10^5^ cells per well were seeded in the upper chambers of the Transwell plates in a serum-free medium. For the invasion assay, the upper chambers were coated with Matrigel (BD Biosciences). Following an additional 24 h of incubation, the cells that had invaded the bottom surface of the membranes were fixed and stained using a 1 mg/mL crystal violet solution. The migrated or invaded cells were then counted using the FSX100 microscope (Olympus, Tokyo, Japan).

### 2.10. MeRIP-Seq

The input group without immunoprecipitation and the m6A IP group incubated with anti-m6A antibodies, then we performed the RNA-seq library generation with NEBNext^®^ Ultra II Directional RNA Library Prep Kit (New England Biolabs, Inc., Ipswich, MA, USA). Sequencing was performed on an Illumina NovaSeq 6000 sequencer (Cloud-Seq Biotech, Shanghai, China). An assessment of quality was executed using the BioAnalyzer 2100 system (Agilent Technologies, Inc., Santa Clara, CA, USA), followed by image analysis. Base read identification and quality control to acquire raw reads (Raw Data). Both biological replicates (R1 and R2) were used for MeRIP-seq analysis. The quality control was performed with the Q30 values in Supplementary Table S3.

The Cutadapt tool (v1.9.3) eliminated 3’ adaptor sequences and low-quality reads. The high-quality reads from the input libraries were then mapped to the reference genome HG19 with the STAR tool, while all libraries’ clean reads were also aligned to the reference genome using the Hisat2 (v2.0.4) software. Methylated sites on lncRNAs, called peaks, were identified using MACS (v 10.10) software [17]. Differential methylation analysis was performed using diffReps to identify sites with differential methylation patterns [18].

### 2.11. Functional Enrichment of Target lncRNAs

The Gene Ontology Project (http://www.geneontology.org accessed on 12 June 2023) was used to provide a controlled vocabulary to describe gene product attributes. The ontology covers three domains: biological process, cellular component, and molecular function. Fisher’s exact test is used to find if there is more overlap between the gene and GO annotation lists than would be expected by chance. The *p*-value denotes the significance of GO terms enrichment in the genes. The lower the *p*-value, the more significant the GO Term is (*p*-value ≤ 0.05 is recommended). Pathway analysis is thefunctional analysis that enriched genes to KEGG pathways. The Fisher *p*-value denotes the significance of the pathway correlated to the conditions. The smaller the *p*-value, the more significant the correlation is (The recommended *p*-value cut-off is 0.05).

To improve the accuracy of the me-RIP sequence data analysis, we used RNA protein interaction prediction (RPISeq) to confirm the association between potential target lncRNAs and METTL3 [19]. In addition, with the SRAMP (Sequence-based RNA Adenosine Methylation Site Predictor) [20], a database for the prediction of m6A modification sites on target RNA sequences, we performed a comprehensive prediction of the m6A sites of our target key lncRNAs.

### 2.12. Statistical Analyses

All biological experiments were performed independently in triplicate. Continuous variables are presented as means with corresponding standard deviations. Analysis of variance (ANOVA) and chi-squared test or non-parametric tests were used to compare data as appropriate. Data analysis was performed using IBM SPSS Statistics 22.0 (SPSS Inc., Chicago, IL, USA) and GraphPad Prism 8.0 (La Jolla, CA, USA). Statistical significance was defined as *p* < 0.05.

## 3. Results

### 3.1. Subsection

#### 3.1.1. Knock-Down of METTL3 Impacted the Migration and Invasion of MC and GC Cells In Vitro

First, we examined the expression of METTL3 mRNA in different generations of MNNG-exposed MC cells, and the results showed that METTL3 was indeed elevated in MC-40 cells compared to control cells (Supplementary Figure S2A). In addition, we measured the METTL3 expression in a GC cohort with 40 paired tumor tissues and adjacent normal tissues. The result verified that METTL3 was up-regulated 3.976-fold in GC tissues (Supplementary Figure S2C). Subsequently, the K–M survival curves with log-rank tests showed that METTL3 overexpression predicted poor outcomes in the GC patients (Supplementary Figure S2D).

**Figure 1:**
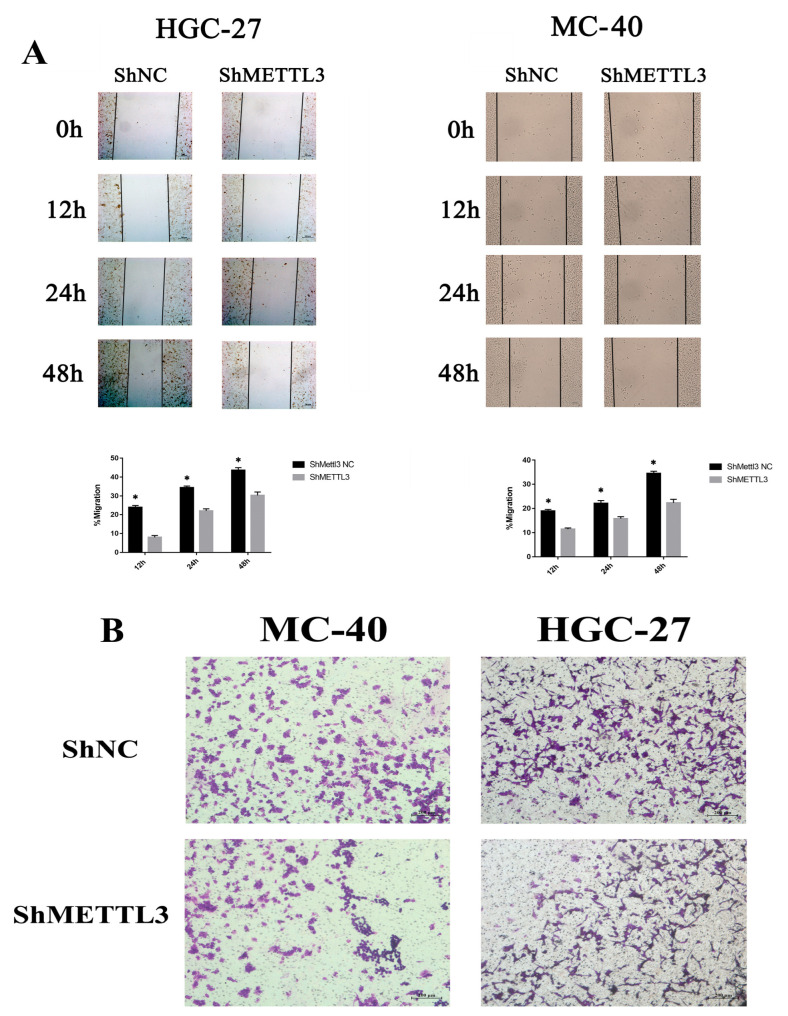
METTL3 downregulation inhibited the migration, and invasion of MC-40 and HGC-27 cell in vitro (**A**) METTL3 knockdown inhibited the migration of MC-40 and HGC-27, * *p* < 0.05. (**B**) METTL3 knockdown inhibited the invasion of MC-40 and HGC-27 cells by Transwell assays.

To assess whether METTL3 has an oncogenic role in MNNG-induced GC, we knocked down the METTL3 in MNNG-induced MC-40 and HGC-27 GC cell lines by shRNA. Scratch wound healing assays demonstrated a significant decrease in MC-40 and HGC-27 GC cell migration ability after METTL3 downregulation (Figure 1A). Moreover, METTL3 silencing impaired the invasion ability of MC-40 and HGC-27 GC cells, as shown by Transwell assays (Figure 1B). Collectively, our findings provide the first evidence demonstrating the oncogenic role of METTL3 in MNNG-induced GC.

#### 3.1.2. Detection of m6A Modifications on lncRNAs in the MC-40 and HGC-27 Cells Using MeRIP-Seq Technologies

**Figure 2:**
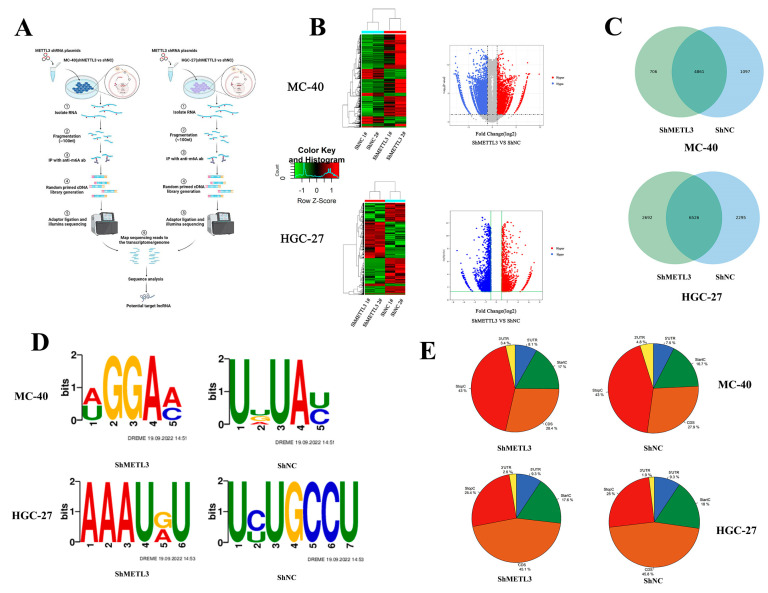
Me-RIP-seq for potential lncRNAs downstream of METTL3 (**A**) Me-RIP-seq screening process for potential lncRNAs downstream of METTL3. (**B**) Heat maps of mRNAs that were differentially transcribed between Sh-METTL3 and matched control cell (FC > 2, *p* < 0.001). (**C**) Numbers of m6A peaks identified on lncRNAs in meRIP-seq from MC-40 and HGC-27 cells. (**D**) The predominant consensus motif was measured by meRIP-seq. (**E**) Distribution of diffpeaks across the length of lncRNA transcripts of MC-40 and HGC-27 cells.

To depict the profile of METTL3-mediated lncRNA m6A modification variation at the transcriptome level in MNNG-induced GC, we performed the MeRIP-seq in MC-40 and HGC-27 cells with stable METTL3 knockdown and control cells (Figure 2A). In total, MeRIP-seq identified 31,778 and 16,167 genes from each group, of which 1432 and 3520 were assigned to lncRNAs (Figure 2B). The composition of sequenced overlapping lncRNAs is illustrated in the panel. In total, m6A-seq identified 4861 and 6526 hub m6A peaks from m6A-modified transcripts in shNC controls and METTL3-deficient MC-40 and HGC-27 cells, respectively (Figure 2C). The predominant consensus motif was identified and depicted. The sequence AUGGAAC (RRACH) motif was highly enriched in the immunopurified RNA samples from METTL3 knockdown MC-40 cells, while the UUGAUAUC motif was enriched in the shNC control MC-40 cells (Figure 2D). A summary of MeRIP-seq data of the two MC-40 and HGC-27 replicates (at gene level) is provided, the distribution of m6A peaks in lncRNA transcripts showed enrichment around the stop codons of the 3’ untranslated regions (UTRs) in both MC-40 and HGC-27 cells (Figure 2E). The top 20 lncRNAs with altered m6A modification levels in both groups are shown in Supplementary Table S4 (MC-40-shMETTL3 vs. MC-40-shNC) and Supplementary Table S5 (HGC-27-shMETTL3 vs. HGC-27-shNC).

#### 3.1.3. Functional Annotation of m6A on lncRNAs Regulated by METTL3

**Figure 3:**
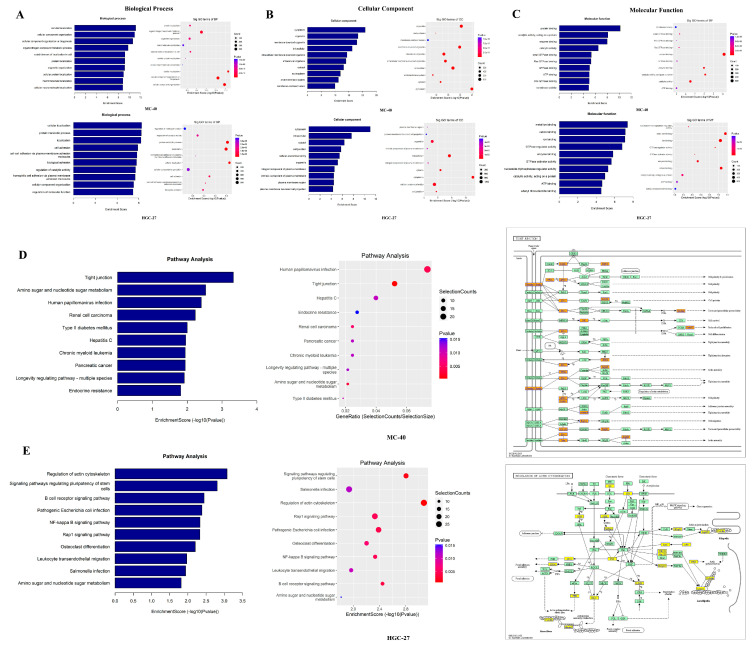
GO and KEGG Pathways analysis of down-regulated METTL3 differentially methylated lncRNAs (**A**) Biological process of downregulated METTL3 differentially methylated lncRNA in MC-40 and HGC-27 cells. (**B**) Cellular component of downregulated METTL3 differentially methylated lncRNA in MC-40 and HGC-27 cells. (**C**) Molecular function of downregulated METTL3 differentially methylated lncRNA in MC-40 and HGC-27 cells. (**D**) Pathway analysis of downregulated METTL3 differentially methylated lncRNA in MC-40 cell. (**E**) Pathway analysis of downregulated METTL3 differentially methylated lncRNA in HGC-27 cell.

To classify the gene function of MNNG-induced malignant transformation and to find its potential pathways, we performed GO analysis and KEGG pathway analysis based on MeRIP-seq data for lncRNAs with different m6A modification levels. As illustrated in Figure 3, we listed the top 10 remarkable GO terms (including the following three domains: biological process, cellular component, and molecular function) for each MC-40 and HGC-27 group. After METTL3 depletion, the most influenced enriched GO terms were cellular localization, cytoplasm, and protein binding for lncRNAs on MC-40 cells (Figure 3A). Similarly, the most influenced enriched GO terms were cellular localization, cytoplasm, and metal ion binding for lncRNAs on HGC-27 cells with METTL3 knockdown (Figure 3A).

As presented, we also displayed ten significant KEGG pathways annotated for each group (Figure 3B,C). Similar to GO analysis, we found many KEGG pathways were related to cellular localization and cytoplasm, such as the tight junction, amino sugar and nucleotide sugar metabolism, regulation of actin cytoskeleton, signaling pathways regulating pluripotency of stem cells, etc. Based on the above results, the GO terms and KEGG pathway analysis implied that MNNG exposure could spark cellular localization change, which might be the critical cellular note variation for malignant transformation.

#### 3.1.4. Screening of Key Downstream lncRNAs Potentially Regulated by METTL3

To understand the regulatory role of METTL3 in MNNG-induced GC and to explore lncRNAs that differ in METTL3-induced m6A peaks, we subsequently focused on lncRNAs in the MeRIP-seq data.

**Figure 4:**
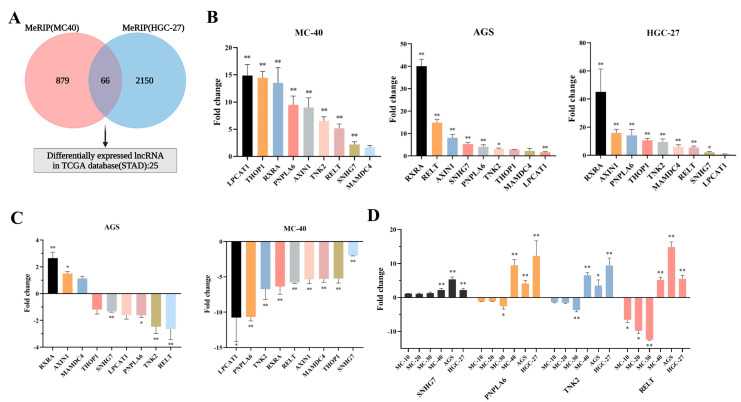
Screening of key downstream lncRNAs potentially regulated by METTL3 (**A**) Screening downstream target lncRNAs potentially regulated by METTL3. (**B**) Nine pivotal lncRNA expressions were detected by qRT-PCR in MC-40 and HGC-27 cells. * *p* < 0.05, ** *p* < 0.01. (**C**) The expression of four key lncRNAs was detected by qRT-PCR in MC-40 and AGS cells with METTL3 knockdown. shMETTL3 vs. shNC, * *p* < 0.05, ** *p* < 0.01. (**D**) The expression of four key lncRNAs was detected by qRT-PCR in different generations of MC cells, GC AGS cells, and HGC-27 cells. * *p* < 0.05, ** *p* < 0.01.

Venn analysis showed that 66 hub-lncRNAs overlapped between METTL3 knockdown by different shRNAs in MC-40 and HGC-27 cells (Figure 4A; Supplementary Table S6). Based on these results, fifteen key lnRNAs were screened for further validation in the TCGA STAD data, MC-40 and two GC cell lines (AGS, HGC-27), including lncRNAs (AXIN1, RELT, LPCAT1, MAMDC4, TNK2, NADSYN1, C12orf60, MC1R, THOP1, PNPLA6, MYH16, PLEKHM1P, SNHG7, RXRA, FGF22) (Table 2). Among the above lncRNAs, nine lncRNAs (PNPLA6, THOP1, RELT, RXRA, TNK2, MAMDC4, LPCAT1, AXIN1, SNHG7) showed a consistent trend of expression in the TCGA STAD data, MC-40 cells (Figure 4B) and two GC cells (HGC-27, AGS).

#### 3.1.5. SNHG7 Was Identified as a Downstream Target of METTL3

**Figure 5:**
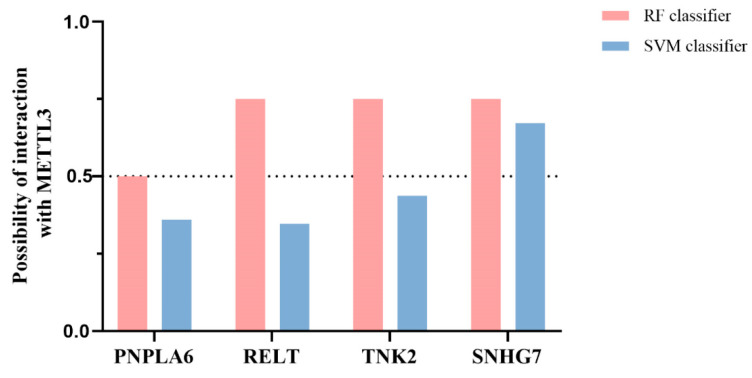
Prediction of interaction probabilities of four key lncRNAs with METTL3 by RPISeq. Probabilities ranging from 0–1; RF > 0.5 and SVM > 0.5 indicate that the corresponding RNA and protein are likely to interact.

**Figure 6:**
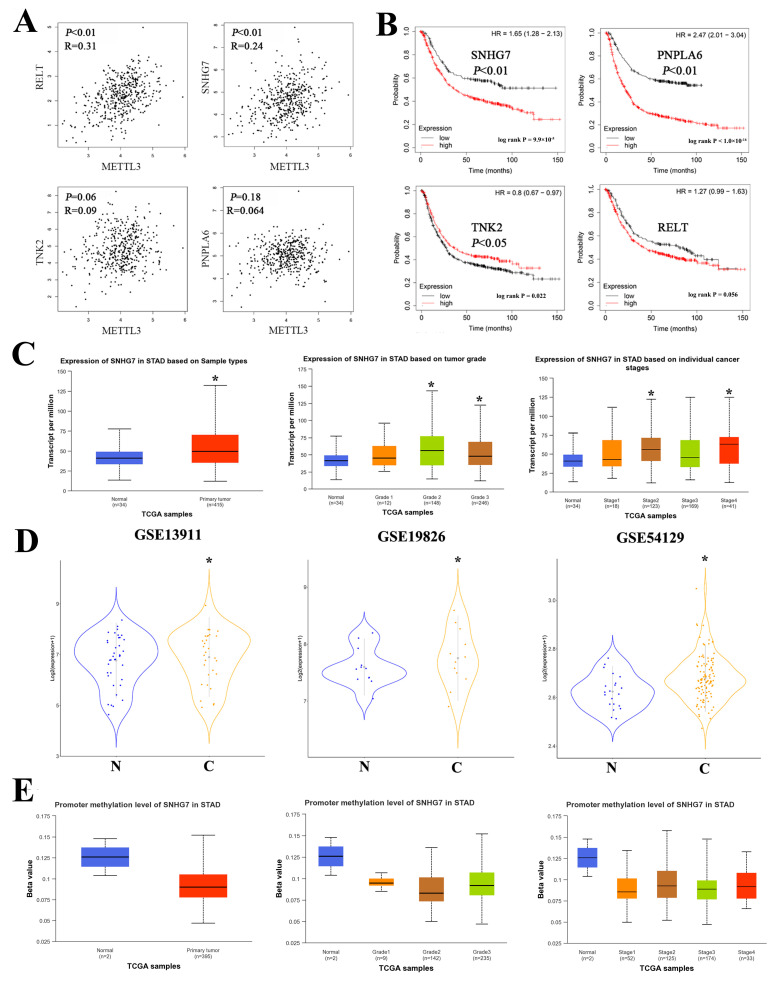
Up-regulation of SNHG7 is associated with progress in GC. (**A**) Correlation analysis of four key lncRNAs and METTL3 expression. (**B**) The Kaplan–Meier estimates survival times in two groups of patients from the GEO GC cohort by different SNHG7 expression levels. (**C**) The SNHG7 expression level of the TCGA STAD cohort. * *p* < 0.05. (**D**) The SNHG7 expression level of the GEO GC cohort. * *p* < 0.05. (**E**) Promoter methylation level of SNHG7 in TCGA STAD cohort.

To further verify the effect of down-regulation of METTL3 on downstream key lncRNAs, qRT-PCR was performed to detect the expression of the nine pivotal lncRNAs in MC-40-shMETTL3, AGS-shMETTL3, and paired control cells (Figure 4C). Notably, only four key lncRNAs (SNHG7, PNPLA6, TNK2, and RELT) expressed a consistent down-regulated trend in both AGS-shMETTL3 and MC-40-shMETTL3 cells compared with control cells AGS-shNC, MC-40-shNC (*p* < 0.05). The SRAMP was then used for m6A site prediction and all four key lncRNAs were found to have m6A sites (Table 3). The expression trends of the four critical lncRNAs in MC cells and GC cells were further analyzed. Only SNHG7 (Small Nucleolar RNA Host Gene 7) expression was progressively up-regulated with MNNG-induced malignant transformation progress. The expression of SNHG7 was elevated in both GC cells AGS (FC = 5.32, *p* < 0.01) and HGC-27 (FC = 2.20, *p* < 0.01) (Figure 4D). In addition, we further analyzed the METTL3-lncRNA interactions by RPI-Seq, and the results revealed that SNHG7 most interacts with METTL3 (RF > 0.5, SVM > 0.5) (Figure 5). Furthermore, we analyzed the correlation between METTL3 expression and target lncRNAs in GC patients from TCGA database and found that RELT and SNHG7 were positively correlated with METTL3 expression (*p* < 0.01) (Figure 6A).

#### 3.1.6. SNHG7 Is Up-Regulated in GC and Associated with a Poor Prognosis

To investigate whether SNHG7 may affect clinical outcomes in GC patients, we first queried TCGA and GEO datasets. SNHG7 expression was significantly elevated in GC patients compared with normal controls (Figure 6C). Three individual GC cohorts (GSE 13911, GSE19826, 54129) sequencing data from the Gene Expression Omnibus (GEO) dataset were used to verify the SNHG7 expression (Figure 6D), and the relevant clinical data were present in Table 4. The elevated SNHG7 expression was also associated with poor prognosis outcomes in GC (Figure 6B). We subsequently examined SNHG7 expression in different stages of GC malignancy and revealed that elevated SNHG7 expression was associated with advanced clinical features, such as TNM stage and tumor grade (Figure 6C). Notably, we also noted that the higher the SNHG7, the lower the DNA methylation of the promoter in patients with advanced GC was (Figure 6E).

## 4. Discussion

Emerging evidence suggests that dysregulated lncRNAs play an important role in GC development and progression [21,22]. The application of next-generation sequencing-based on antibody enrichment, which involves co-precipitation incubation of fragmented RNA with m6A antibodies followed by high-throughput sequencing [23], supports the potential edge of MeRIP-seq in analyzing specific m6A modifications in lncRNAs [24]. Based on the MeRIP-seq data, the present study identified a panel of lncRNAs aberrantly expressed in the malignant transformation of GES-1 cells exposed to MNNG.

First, we confirmed that long-term MNNG exposure increased the METTL3 mRNA expression profile of MNNG-induced malignant cells by qRT-PCR. Furthermore, qRT-PCR results from the TCGA STAD cohort and an individual GC cohort also verified that METTL3 expression was significantly elevated in GC patients, which was in line with previous study [25]. Subsequently, K–M survival curves with log-rank tests showed that METTL3 overexpression predicted poor outcomes in the GC patients, indicating the potential oncogenic role of METTL3 in GC carcinogenesis.

In this context, to identify METTL3-regulate lnRNAs through m6A modification, we performed peak calling on MeRIP-seq data and identified 4861 and 6526 hub m6A peaks that were assigned to lncRNAs that were specifically crosslinked to METTL3. Comparing MC-40-shMETTL3 with its control cells, the m6A modification levels of 1434 lncRNA transcripts were found to be significantly altered (FC > 2, *p* < 0.01), of which 945 were significantly down-regulated, and 489 were significantly up-regulated; whereas, in the HGC-27-shMETTL3 compared with its corresponding control cells, the m6A modification levels of 3520 lncRNA transcripts were significantly altered (FC > 2, *p* < 0.01), of which 2251 lncRNA transcripts were significantly down-regulated and 1269 lncRNA transcripts were significantly up-regulated. Moreover, we found the consensus sequence AUGGAAC (RRACH) motif was highly enriched MC-40 cell with METTL3 knockdown, which resembles the common m6A motif described in severe human diseases [26,27,28]. Furthermore, the results showed that m6A peaks were significantly related to gene locations in both MC-40 and HGC-27 cells near the stop codons of the 3’ untranslated regions (UTRs), which were different from the sequencing results of GC tissue samples (near the middle of the 5’ UTRs) [26] 

Bioinformatics analysis was applied to predict the potential bio-functions and related pathways of lncRNAs by dysregulated m6A modification in MNNG-induced GC. Then, we mainly focused on those key lncRNAs whose m6A modification levels were significantly down-regulated, so we selected intersecting lncRNAs from the METTL3 down-regulation groups and subjected them to GO analysis and KEGG pathway analysis. According to the GO results, more than 1/3 of the GO terms are related to cellular localization with changes in cell structure, such as cytoplasm, cellular protein localization, and metal ion binding. We have also identified links between key KEGG pathways and changes in cellular material metabolism and intercellular structure, such as tight junctions, amino acid sugar, nucleotide sugar metabolism, actin cytoskeleton regulation, and stem cell pluripotency regulation signaling pathways, which were reported to be associated with GC growth and metastasis [29,30].

To further confirm the association of METTL3 with the proposed target lncRNAs, we analyzed the publicly available GC data and verified the expression levels of critical lncRNAs by using qRT-PCRs in MC and GC cells. Nine lncRNAs (PNPLA6, THOP1, RELT, RXRA, TNK2, MAMDC4, LPCAT1, AXIN1, SNHG7) were identified for further screening by qRT-PCR. Four key lncRNAs (SNHG7, PNPLA6, TNK2, and RELT) were selected with a consistent down-regulated trend in both GC and MC cells. To highlight the target lncRNAs modified by METTL3, we compared the m6A binding sites identified by SRAMP for the above lncRNAs and found that about four key lncRNAs overlapped with the m6A sites, implying that METTL3 modifies these transcripts. We further evaluated the trends of the four key lncRNA qRT-PCR expression levels in different generations of MC cells and GC cells, and the results showed that only SNHG7 expression showed a high degree of consistency. METTL3-lncRNA interaction and correlation analysis further validated the accuracy of the screening results, which revealed that SNHG7 most interacts with METTL3 and positively significantly correlated with METTL3 expression.

SNHG7 was first reported by Chaudhry in 2013 [31] and previous studies have demonstrated that SNHG7 is upregulated and plays an oncogenic role in cancers [32,33], including bladder cancer [34], breast cancer [35], colorectal cancer [36], and gastric cancer [37], and positively correlates with advanced clinicopathological features and prognosis. In addition, a recent study has revealed an important regulatory mechanism by which SNHG7 mediates cisplatin resistance through the miR-34a/LDHA-glycolysis axis in GC [38]. Based on in vivo and in vitro experiments, Wang et al. revealed that lncRNA SNHG7 partially contributes to the proliferation and apoptosis of GC cells through the regulation of p15 and p16 expressions [39]. Although several studies have explored the potential functions and molecular mechanisms of SNHG7 in GC progression, little attention has been paid to the mechanism of its role in malignant transformation induced by environmental chemical carcinogens. In addition, we noted that the degree of DNA methylation of the SNHG7 promoter was negatively correlated with tumor progression in GC patients and that there appeared to be a feedback regulatory relationship between the level of m6A methylation of SNHG7 and the level of DNA methylation. Den et al. have also characterized METTL3-mediated m6A formation, which leads to DNA demethylation nearby, resulting in increased chromatin accessibility and expression of host genes, revealing for the first time that RNA methylation regulates DNA methylation [40]. In addition, it was recently found that DNA methylation can affect m6A modification by regulating m6A demethylase gene expression so that m6A demethylase feedback regulates DNA methylation [41].

Here, we investigated the up-regulation of SNHG7 expression in different GC cells and GC tissues. More importantly, we observed that SNHG7 was progressively up-regulated during gastric carcinogenesis by MNNG exposure. In contrast, the knockdown of the methylation enzyme METTL3 significantly affected SNHG7 expression.

A recent multivariate Cox proportional hazards analysis showed that high SNHG7 expression was an independent adverse prognostic factor affecting overall survival in GC patients [37]. Wang et al. also demonstrated that the expression levels of SNHG7 were upregulated in 68 gastric cancer tissues and five gastric cancer cell lines (BGC823, MGC803, SGC7901, N87, and AGS) [42]. In line with these studies, we also found that elevated SNHG7 expression was associated with advanced clinical features, such as TNM stage and tumor grade, and poor prognostic outcomes, suggesting its potential as a therapeutic target and effective biomarker. Taken together, interference with the expression of SNHG7 might provide a critical theoretical basis for inverting the malignant transformation of gastric cancer in clinical practice.

In this study, the Me-RIP-seq was applied for the first time to map the lncRNA profiles with significantly altered levels of m6A modification during the malignant transformation of MNNG, and combined with the TCGA database, different cellular expression profiles and bioinformatics analyses, we precisely targeted SNHG7, a critical lncRNA regulated by METTL3 through m6A modification.

## 5. Conclusions

In summary, taken together, our findings implied that METTL3 regulated the m6A methylation level of lncRNA SNHG7, resulting in changes in its expression level, suggesting that SNHG7 may be an essential prognostic factor in the progression of GC. However, further studies are needed to explore the deep mechanism and biological function of SNHG7 in GC carcinogenesis due to MNNG exposure.

## Figures and Tables

**Table 1:** Primer sequences of target genes.

Gene	Primer	Sequences
PLEKHM1P	Forward primer	5′-CCAAGACGATGCCTCAGTGATTCC-3′
	Reverse primer	5′-CAACACGGAACCTATGCGGAGAC-3′
MYH16	Forward primer	5′-GAAGGAGCACCAGGACCGAATTG-3′
	Reverse primer	5′-ACCTTGGCGTTGGCTTCTGATAAG-3′
PNPLA6	Forward primer	5′-GTCGGTTTGCTCCATCCCTTAGTC-3′
	Reverse primer	5′-GAGCCTCCATCGGTTGATTCCAG-3′
FGF22	Forward primer	5′-ATCTGGCAGGTGAGGACAAGGAG-3′
	Reverse primer	5′-GATGAAAGCGGTGGGAAGGACAG-3′
THOP1	Forward primer	5′-TCGGCAAGTTCTACCTGGACCTG-3′
	Reverse primer	5′-GTGGCTGAGTGAGACTGGAAACG-3′
RELT	Forward primer	5′-TAGCCGCCACTACTCCTGTTCC-3′
	Reverse primer	5′-GGACCAGAGCCTTAGCCTGAGAG-3′
MC1R	Forward primer	5′-ACCCTTAGGAGGCAGCAGACAC-3′
	Reverse primer	5′-CACGGTCATTGGAAGTAGGCTCAG-3′
C12orf60	Forward primer	5′-CACACTGACACTGGCACACCTG-3′
	Reverse primer	5′-GATCACACCGAGGCTTGGAGAATG-3′
NADSYN1	Forward primer	5′-CCTTGGCTCGCTTCTTCCTTGG-3′
	Reverse primer	5′-ACTGGTGTTGCTTGTGTGTTTTGTG-3′
RXRA	Forward primer	5′-AATGCTGCCTTCTGCCTTCTCAAG-3′
	Reverse primer	5′-CACCAACTCACTCCACCAATACCTG-3′
TNK2	Forward primer	5′-TAACTCATCGGCTACTCAGGAAGGG-3′
	Reverse primer	5′-ACACGGCGGTCCAGTATGATAGG-3′
MAMDC4	Forward primer	5′-GACACAAGCCCAGACGCACTAC-3′
	Reverse primer	5′-CCACTGCCTCAAACACCACCTG-3′
LPCAT1	Forward primer	5′-GCTGCCGTATGCTGATCCTAACC-3′
	Reverse primer	5′-CACCTCTTCCCAAAGCCATCTGAC-3′
AXIN1	Forward primer	5′-TCCATCCACTGAGAACCACTGAGG-3′
	Reverse primer	5′-TGACAAGAGGACACAGCGAGGAG-3′
SNHG7	Forward primer	5′-TGCTCACTGGAGATGACACG-3′
	Reverse primer	5′-TCCATCACAGGCGAAGTCAC-3′
GAPDH	Forward primer	5′-TCCTCTGACTTCAACAGCGACAC-3′
	Reverse primer	5′-CACCCTGTTGCTGTAGCCAAATTC-3′

**Table 2:** 15 key lncRNAs validated for TCGA STAD data, MC-40, and GC cells.

Gene	Regulation			
	MC-40	AGS	HGC-27	TCGA Expression Type
PLEKHM1P	Down	Down *	Up *	Up *
MYH16	Up	Down	Down *	Up *
**PNPLA6**	Up *	Up *	Up *	Up *
FGF22	Up *	Down *	Up *	Down *
**THOP1**	Up *	**Up**	Up *	Up *
**RELT**	Up *	Up *	Up *	Up *
MC1R	Down *	Up	Up *	Up *
C12orf60	Down	Up *	Up *	Up *
NADSYN1	Up	Down *	Up *	Up *
**RXRA**	Up *	Up *	Up *	Down *
**TNK2**	Up *	Up *	Up *	Up *
**MAMDC4**	**Up**	**Up**	Up *	Up *
**LPCAT1**	Up *	Up *	**Up**	Up *
**AXIN1**	Up *	Up *	Up *	Up *
**SNHG7**	Up *	Up *	Up *	Up *

Up: lncRNA expression up-regulated; Down: lncRNA expression down-regulated; * *p* < 0.05 compared with GES-1 cells and adjacent control tissues.

**Table 3:** m6A site confidence prediction of 4 key lncRNAs.

Gene	m6A Site Confidence (Number)				
	Low	Moderate	High	Very High	Summary
RELT	4	6	7	3	20
SNHG7	1	4	1	0	6
TNK2	7	8	10	13	38
PNPLA6	1	1	2	0	4

**Table 4:** The clinical feature of GEO GC cohorts.

Geo Dataset	Public Year	Country	Platform	Samples	N
GSE54129	2017	China	GPL570	GC	111
				Normal Tissue	21
GSE13911	2008	Italy	GPL570	GC	38
				Normal Tissue	31
GSE19826	2010	China	GPL570	GC	12
				Normal Tissue	12

## Data Availability

The datasets generated and analyzed during the current study are available in the [TCGA] repository (http://cancergenome.nih.gov accessed on 12 June 2023). Data will be made available on request.

## Supplementary Materials

The following supporting information can be downloaded at: https://www.mdpi.com/article/10.3390/toxics11110944/s1, Figure S1: Knock-down of METTL3 in MC and GC cells. Figure S2: METTL3 expression in GC cells and patients. Table S1: Clinical features of GC patients; Table S2: Primer sequences of ShMETT3 and ShNC; Table S3: MeRIP-seq Q30 controlled quality control results of all reads; Table S4: MC-40 knockdown METTL3 (Sh-METTL3) and control cell group (Sh-NC) differential m6A Peak association analysis (top 20 m6A up-regulated, down-regulated); Table S5: HGC-27 knockdown METTL3 (Sh-METTL3) and control cell group (Sh-NC) differential m6A Peak association analysis (top 20 m6A up-regulated, down-regulated); Table S6: 66 hub-lncRNAs overlapped between METTL3 knockdown by different shRNAs in MC-40 and HGC-27 cells.

## References

[1] Siegel R.L., Miller K.D., Jemal A. (2020). Cancer statistics. CA Cancer J. Clin.

[2] Karimi P., Islami F., Anandasabapathy S., Freedman N.D., Kamangar F. (2014). Gastric cancer: Descriptive epidemiology, risk factors, screening, and prevention. Cancer Epidemiol. Biomark. Prev..

[3] Lopez M.J., Carbajal J., Alfaro A.L., Saravia L.G., Zanabria D., Araujo J.M., Quispe L., Zevallos A., Buleje J.L., Cho C.E. (2023). Characteristics of gastric cancer around the world. Crit. Rev. Oncol. Hematol..

[4] Gunes-Bayir A., Guler E.M., Bilgin M.G., Ergun I.S., Kocyigit A., Dadak A. (2022). Anti-Inflammatory and Antioxidant Effects of Carvacrol on N-Methyl-N’-Nitro-N-Nitrosoguanidine (MNNG) Induced Gastric Carcinogenesis in Wistar Rats. Nutrients.

[5] Wang Q., Chen C., Ding Q., Zhao Y., Wang Z., Chen J., Jiang Z., Zhang Y., Xu G., Zhang J. (2020). METTL3-mediated m(6)A modification of HDGF mRNA promotes gastric cancer progression and has prognostic significance. Gut.

[6] Jia Y., Yan Q., Zheng Y., Li L., Zhang B., Chang Z., Wang Z., Tang H., Qin Y., Guan X.Y. (2022). Long non-coding RNA NEAT1 mediated RPRD1B stability facilitates fatty acid metabolism and lymph node metastasis via c-Jun/c-Fos/SREBP1 axis in gastric cancer. J. Exp. Clin. Cancer Res..

[7] Liu H.T., Zou Y.X., Zhu W.J., Sen L., Zhang G.H., Ma R.R., Guo X.Y., Gao P. (2022). lncRNA THAP7-AS1, transcriptionally activated by SP1 and post-transcriptionally stabilized by METTL3-mediated m6A modification, exerts oncogenic properties by improving CUL4B entry into the nucleus. Cell Death Differ..

[8] Zhang H.M., Qi F.F., Wang J., Duan Y.Y., Zhao L.L., Wang Y.D., Zhang T.C., Liao X.H. (2022). The m6A Methyltransferase METTL3-Mediated N6-Methyladenosine Modification of DEK mRNA to Promote Gastric Cancer Cell Growth and Metastasis. Int. J. Mol. Sci..

[9] Sun Y., Li S., Yu W., Zhao Z., Gao J., Chen C., Wei M., Liu T., Li L., Liu L. (2020). N(6)-methyladenosine-dependent pri-miR-17-92 maturation suppresses PTEN/TMEM127 and promotes sensitivity to everolimus in gastric cancer. Cell Death Dis..

[10] Liu N., Dai Q., Zheng G., He C., Parisien M., Pan T. (2015). N(6)-methyladenosine-dependent RNA structural switches regulate RNA-protein interactions. Nature.

[11] Liu H., Xu Y., Yao B., Sui T., Lai L., Li Z. (2020). A novel N6-methyladenosine (m6A)-dependent fate decision for the lncRNA THOR. Cell Death Dis..

[12] Zhang J., Guo S., Piao H.Y., Wang Y., Wu Y., Meng X.Y., Yang D., Zheng Z.C., Zhao Y. (2019). ALKBH5 promotes invasion and metastasis of gastric cancer by decreasing methylation of the lncRNA NEAT1. J. Physiol. Biochem..

[13] He X., Wu W., Lin Z., Ding Y., Si J., Sun L.M. (2018). Validation of the American Joint Committee on Cancer (AJCC) 8th edition stage system for gastric cancer patients: A population-based analysis. Gastric Cancer.

[14] Chandrashekar D.S., Bashel B., Balasubramanya S.A.H., Creighton C.J., Ponce-Rodriguez I., Chakravarthi B., Varambally S. (2017). UALCAN: A Portal for Facilitating Tumor Subgroup Gene Expression and Survival Analyses. Neoplasia.

[15] Gyorffy B. (2023). Discovery and ranking of the most robust prognostic biomarkers in serous ovarian cancer. Geroscience.

[16] Liu T., Yang S., Sui J., Xu S.Y., Cheng Y.P., Shen B., Zhang Y., Zhang X.M., Yin L.H., Pu Y.P. (2020). Dysregulated N6-methyladenosine methylation writer METTL3 contributes to the proliferation and migration of gastric cancer. J. Cell. Physiol..

[17] Zhang Y., Liu T., Meyer C.A., Eeckhoute J., Johnson D.S., Bernstein B.E., Nusbaum C., Myers R.M., Brown M., Li W. (2008). Model-based analysis of ChIP-Seq (MACS). Genome Biol..

[18] Shen L., Shao N.Y., Liu X., Maze I., Feng J., Nestler E.J. (2013). diffReps: Detecting differential chromatin modification sites from ChIP-seq data with biological replicates. PLoS ONE.

[19] Muppirala U.K., Honavar V.G., Dobbs D. (2011). Predicting RNA-protein interactions using only sequence information. BMC Bioinform..

[20] Zhou Y., Zeng P., Li Y.H., Zhang Z., Cui Q. (2016). SRAMP: Prediction of mammalian N6-methyladenosine (m6A) sites based on sequence-derived features. Nucleic Acids Res..

[21] Duan F., Li Y., Feng Y., Niu G., Chai J., Wang K. (2023). Increased lncRNA AFAP1-AS1 expression predicts poor prognosis in gastric cancer: Evidence from published studies and followed up verification. Cancer Med..

[22] Jiang W., Meng K., Yang T. (2023). Long non-coding RNA PROX1-AS1 promotes the proliferation and migration in gastric cancer by epigenetically activating FGFR1. Panminerva Med..

[23] Meyer K.D., Saletore Y., Zumbo P., Elemento O., Mason C.E., Jaffrey S.R. (2012). Comprehensive analysis of mRNA methylation reveals enrichment in 3’ UTRs and near stop codons. Cell.

[24] Krusnauskas R., Stakaitis R., Steponaitis G., Almstrup K., Vaitkiene P. (2023). Identification and comparison of m6A modifications in glioblastoma non-coding RNAs with MeRIP-seq and Nanopore dRNA-seq. Epigenetics.

[25] Yue B., Song C., Yang L., Cui R., Cheng X., Zhang Z., Zhao G. (2019). METTL3-mediated N6-methyladenosine modification is critical for epithelial-mesenchymal transition and metastasis of gastric cancer. Mol. Cancer.

[26] Sang L., Sun L., Wang A., Zhang H., Yuan Y. (2020). The N6-Methyladenosine Features of mRNA and Aberrant Expression of m6A Modified Genes in Gastric Cancer and Their Potential Impact on the Risk and Prognosis. Front. Genet..

[27] Zhang J., Piao H.Y., Wang Y., Meng X.Y., Yang D., Zhao Y., Zheng Z.C. (2020). To Develop and Validate the Combination of RNA Methylation Regulators for the Prognosis of Patients with Gastric Cancer. OncoTargets Ther..

[28] He X., Shu Y. (2019). RNA N6-methyladenosine modification participates in miR-660/E2F3 axis-mediated inhibition of cell proliferation in gastric cancer. Pathol. Res. Pract..

[29] Lu Y.X., Ju H.Q., Liu Z.X., Chen D.L., Wang Y., Zhao Q., Wu Q.N., Zeng Z.L., Qiu H.B., Hu P.S. (2018). ME1 Regulates NADPH Homeostasis to Promote Gastric Cancer Growth and Metastasis. Cancer Res..

[30] Pajuelo-Lozano N., Alcala S., Sainz B., Perona R., Sanchez-Perez I. (2020). Targeting MAD2 modulates stemness and tumorigenesis in human Gastric Cancer cell lines. Theranostics.

[31] Chaudhry M.A. (2013). Expression pattern of small nucleolar RNA host genes and long non-coding RNA in X-rays-treated lymphoblastoid cells. Int. J. Mol. Sci..

[32] Zhou Y., Tian B., Tang J., Wu J., Wang H., Wu Z., Li X., Yang D., Zhang B., Xiao Y. (2020). SNHG7: A novel vital oncogenic lncRNA in human cancers. Biomed. Pharmacother..

[33] Najafi S., Ghafouri-Fard S., Hussen B.M., Jamal H.H., Taheri M., Hallajnejad M. (2021). Oncogenic Roles of Small Nucleolar RNA Host Gene 7 (SNHG7) Long Noncoding RNA in Human Cancers and Potentials. Front. Cell Dev. Biol..

[34] Xu C., Zhou J., Wang Y., Wang A., Su L., Liu S., Kang X. (2019). Inhibition of malignant human bladder cancer phenotypes through the down-regulation of the long non-coding RNA SNHG7. J. Cancer.

[35] Sun X., Huang T., Liu Z., Sun M., Luo S. (2019). LncRNA SNHG7 contributes to tumorigenesis and progression in breast cancer by interacting with miR-34a through EMT initiation and the Notch-1 pathway. Eur. J. Pharmacol..

[36] Li Y., Zeng C., Hu J., Pan Y., Shan Y., Liu B., Jia L. (2018). Long non-coding RNA-SNHG7 acts as a target of miR-34a to increase GALNT7 level and regulate PI3K/Akt/mTOR pathway in colorectal cancer progression. J. Hematol. Oncol..

[37] Zhang Y., Yuan Y., Zhang Y., Cheng L., Zhou X., Chen K. (2020). SNHG7 accelerates cell migration and invasion through regulating miR-34a-Snail-EMT axis in gastric cancer. Cell Cycle.

[38] Pei L.J., Sun P.J., Ma K., Guo Y.Y., Wang L.Y., Liu F.D. (2021). LncRNA-SNHG7 interferes with miR-34a to de-sensitize gastric cancer cells to cisplatin. Cancer Biomark.

[39] Wang M.W., Liu J., Liu Q., Xu Q.H., Li T.F., Jin S., Xia T.S. (2017). LncRNA SNHG7 promotes the proliferation and inhibits apoptosis of gastric cancer cells by repressing the P15 and P16 expression. Eur. Rev. Med. Pharmacol. Sci..

[40] Deng S., Zhang J., Su J., Zuo Z., Zeng L., Liu K., Zheng Y., Huang X., Bai R., Zhuang L. (2022). RNA m(6)A regulates transcription via DNA demethylation and chromatin accessibility. Nat. Genet..

[41] Zhou L., Tian S., Qin G. (2019). RNA methylomes reveal the m(6)A-mediated regulation of DNA demethylase gene SlDML2 in tomato fruit ripening. Genome Biol..

[42] Bian Z., Ji W., Xu B., Huang W., Jiao J., Shao J., Zhang X. (2020). The role of long noncoding RNA SNHG7 in human cancers (Review). Mol. Clin. Oncol..

