# Supplementary material for: Depicting the Profile of METTL3-Mediated lncRNA m6A Modification Variants and Identified SNHG7 as a Prognostic Indicator of MNNG-Induced Gastric Cancer
Source: Toxics. 2023 Nov 20;11(11):944. doi: 10.3390/toxics11110944 (PMC10674297; doi:10.3390/toxics11110944)
Supplement: Supplementary file 1 [file toxics-11-00944-s001.zip › toxics-2706948-supplementary.pdf]

Supplementary Table 1 Clinical features of GC patients

Clinical Features	Group	Case (N=40)
**Gender**	**Female**	**4**
	**Male**	**36**
**Age**	**≤65**	**33**
	**> 65**	**7**
**Tumor Grade**	**G1**	**5**
	**G2**	**6**
	**G3**	**7**
	**unknown**	**22**
**Serum bio-feature**	**CEA**	**18.28±54.87**
	**CA199**	**22.07±56.73**

Supplementary 2 Primer sequences of ShMETT3 and ShNC

RNAs	Primer sequences (5' to 3')
ShMETTL3 Top strand:	GATCCGCAAGTATGTTCACTATGAAATTCAAGAGATTTCATAGTGAACATACTTGCTTTTTTG
ShMETTL3 Bottom strand:	AATTCAAAAAAGCAAGTATGTTCACTATGAAATCTCTTGAATTTCATAGTGAACATACTTGCG
ShNC Top strand:	GATCCGTTCTCCGAACGTGTCACGTAATTCAAGAGATTACGTGACACGTTCGGAGAATTTTTTC
ShNC Bottom strand:	AATTGAAAAAATTCTCCGAACGTGTCACGTAATCTCTTGAATTACGTGACACGTTCGGAGAACG

Supplementary Table 3 MeRIP-seq Q30 controlled quality control results of all reads.

Sample	Raw Reads	Clean Reads	Clean Ratio	Mapped Reads	Mapped Ratio	Q30
40-MC ShMETTL3 1. IP	84,594,658	84,590,842	99.99%	77,568,157	91.70%	87.30%
40-MC ShMETTL3 2. IP	83,473,990	83,469,782	99.99%	75,685,449	90.67%	89.00%
MC-40 ShNC1.IP	84,622,750	84,617,590	99.99%	77,768,560	91.91%	89.03%
MC-40 ShNC2.IP	83,918,590	83,914,050	99.99%	77,022,239	91.79%	87.63%
40-MC ShMETTL3 1. Input	85,104,868	85,104,518	99.99%	78,070,702	91.74%	86.96%
40-MC ShMETTL3 2. Input	67,134,618	67,134,336	99.99%	61,766,319	92.00%	85.64%
MC-40 ShNC1. Input	98,415,540	98,415,234	99.99%	90,334,431	91.79%	86.90%
MC-40 ShNC2. Input	73,437,368	73,436,978	99.99%	66,683,558	90.80%	85.28%
HGC-27 ShMETTL3 1. IP	83,224,296	83,085,150	99.83%	64,791,934	77.98%	89.64%
HGC-27 ShMETTL3 2. IP	68,345,412	68,164,614	99.74%	50,991,734	74.81%	90.64%
HGC-27 ShNC1.IP	63,956,998	63,888,662	99.89%	51,001,194	79.83%	88.41%
HGC-27 ShNC2.IP	70,445,272	70,363,832	99.88%	55,983,475	79.56%	90.26%
HGC-27 ShMETTL3 1. Input	65,239,038	65,237,436	99.99%	58,578,740	89.79%	87.25%

HGC-27 ShMETTL3 2. Input	61,128,492	61,126,958	99.99%	54,526,522	89.20%	90.49%
HGC-27 ShNC1. Input	68,304,326	68,303,244	99.99%	60,745,894	88.94%	89.85%
HGC-27 ShNC2. Input	66,587,438	66,586,314	99.99%	59,215,526	88.93%	87.45%

Supplementary Table 4 MC-40 knockdown METTL3 (Sh-METTL3) and control cell group (Sh-NC) differential m6A Peak association analysis (top 20 m6A up-regulated, down-regulated)

**Bed format information of differentially methylated RNA sites**					**Annotation**		**Comparison information**			
**Chrom**	**Tx-Start**	**Tx-End**	**Peak-ID**	**Score**	**Transcript id**	**Gene Name**	**Fold change**	**P-value**	**FDR**	**Regulation**
chr9	42712721	42712860	diffreps_peak_890064	8.580353403	uc004acm.3	*CBWD5*	511.3	<0.01	<0.01	up
chr19	5714913	5714940	diffreps_peak_434683	8.405763605	ENST00000590728	*LONP1*	382.1	<0.01	<0.01	up
chr5	77417545	77417697	diffreps_peak_718611	8.455305125	ENST00000517561	*AP3B1*	341.6	<0.01	<0.01	up
chr7	27244741	27245360	diffreps_peak_802411	8.525397346	NR_037843	*HOTTIP*	331.4	<0.01	<0.01	up
chr2	231914041	231914434	diffreps_peak_537594	8.471231696	ENST00000463834	*C2orf72*	236.4	<0.01	<0.01	up
chr1	119605476	119605820	diffreps_peak_53361	8.464151809	NR_126447	*WARS2-IT1*	216.4	<0.01	<0.01	up
chr11	64793581	64793987	diffreps_peak_156987	8.383371263	ENST00000605239	*AP000436.4*	201.6	<0.01	<0.01	up
chr10	95434121	95434400	diffreps_peak_120454	8.268578543	ENST00000460752	*FRA10AC1*	186.5	<0.01	<0.01	up
chr1	100133621	100134120	diffreps_peak_47247	7.920845368	ENST00000605613	*PALMD*	171.5	<0.01	<0.01	up
chr10	988419	988683	diffreps_peak_96189	7.578935618	NR_120629	*LOC101927762*	161.4	<0.01	<0.01	up
chr6	159639328	159639400	diffreps_peak_790884	7.948831058	ENST00000480856	*FNDC1*	161.1	<0.01	<0.01	up
chr17	5151141	5151364	diffreps_peak_362384	7.374488841	ENST00000573772	*RP11-333E1.1*	156.5	<0.01	<0.01	up
chr8	103899201	103899375	diffreps_peak_869414	7.377189108	ENST00000517581	*AZIN1*	156.4	<0.01	<0.01	up
chr7	43967661	43967710	diffreps_peak_807284	7.190614824	ENST00000440899	*UBE2D4*	151.3	<0.01	<0.01	up
chr4	135248221	135248660	diffreps_peak_689186	6.96103457	ENST00000508165	*PES1P1*	146.4	<0.01	<0.01	up

chr7	127938261	127938540	diffreps_peak_834685	6.997274805	NR_046216	*MGC27345*	146.2	<0.01	<0.01	up
chr6	3179837	3179940	diffreps_peak_748278	7.757328689	ENST00000404155	*TUBB2BP1*	143.2	<0.01	<0.01	up
chr9	138076981	138077420	diffreps_peak_921113	6.736270467	NR_046107	*LOC401557*	141.4	<0.01	<0.01	up
chr15	21224716	21224820	diffreps_peak_278524	6.736270467	ENST00000561158	*KIAA0125P2*	141.4	<0.01	<0.01	up
chr19	28192398	28192460	diffreps_peak_450600	6.751321321	ENST00000588027	*LINC00662*	141.3	<0.01	<0.01	up
chr14	60865741	60866067	diffreps_peak_259534	8.351724527	ENST00000555315	*GNRHR2P1*	1178.8	<0.01	<0.01	down
chr11	61909081	61909217	diffreps_peak_154168	8.335201539	ENST00000528375	*INCENP*	372.1	<0.01	<0.01	down
chr22	50172801	50173958	diffreps_peak_604233	8.732471971	uc011ari.1	*LOC90834*	260.2	<0.01	<0.01	down
chr19	640821	641460	diffreps_peak_427949	9.130349886	ENST00000591390	*FGF22*	229.6	<0.01	<0.01	down
chr12	53699479	53699560	diffreps_peak_200770	8.305267111	ENST00000550199	*C12orf10*	178.3	<0.01	<0.01	down
chr5	180629587	180629940	diffreps_peak_746599	7.749912175	ENST00000502812	*CTC-338M12.6*	175.5	<0.01	<0.01	down
chr12	111081160	111081540	diffreps_peak_217398	7.55458863	ENST00000460357	*TCTN1*	166.7	<0.01	<0.01	down
chr19	65661	66460	diffreps_peak_427482	9.855266778	ENST00000606872	*WASH5P*	164.9	<0.01	<0.01	down
chr20	34164088	34164205	diffreps_peak_556406	11.62527444	NR_119376	*FER1L4*	163.3	<0.01	<0.01	down
chr14	73730161	73730490	diffreps_peak_265127	7.306160124	ENST00000555700	*PAPLN*	161.1	<0.01	<0.01	down
chr7	134132401	134132640	diffreps_peak_837938	7.722035668	ENST00000434222	*AKR1B1*	159.3	<0.01	<0.01	down
chr17	61917461	61917644	diffreps_peak_395525	7.508511186	ENST00000581353	*RN7SL805P*	159	<0.01	<0.01	down
chr2	25472486	25472572	diffreps_peak_482757	7.317150923	ENST00000470983	*DNMT3A*	158.3	<0.01	<0.01	down
chr16	28711861	28712221	diffreps_peak_331423	6.948060532	ENST00000564603	*CDC37P1*	152.1	<0.01	<0.01	down
chr1	31198566	31198898	diffreps_peak_21151	6.462661971	ENST00000443076	*MATN1-AS1*	139	<0.01	<0.01	down
chr22	24826101	24826344	diffreps_peak_588785	6.420496072	NR_028484	*ADORA2A-AS1*	138.7	<0.01	<0.01	down
chr2	217082581	217082900	diffreps_peak_532769	8.13078575	NR_037701	*PKI55*	136.5	<0.01	<0.01	down
chr5	150414056	150414060	diffreps_peak_737385	7.165491001	ENST00000517329	*TNIP1*	135.6	<0.01	<0.01	down
chr1	241587591	241587849	diffreps_peak_92008	6.333892394	ENST00000444330	*RP11-527D7.1*	134.6	<0.01	<0.01	down
chr9	90583441	90584000	diffreps_peak_897054	6.250822109	ENST00000603475	*CDK20*	133.4	<0.01	<0.01	down

Differentially methylated RNA sites for: Case vs Control； Fold change cut-off: 2； P-value cut-off: 0.01； FDR: the corrected p-value between two groups；Regulation: up or down in test group； Transcript id: the unique

identifier describing the predicted lncRNA assigned by stringtie; Gene Name: the gene id assigned by stringtie

Supplementary Table 5 HGC-27 knockdown METTL3 (Sh-METTL3) and control cell group (Sh-NC) differential m6A Peak association analysis (top 20 m6A up-regulated, down-regulated)

**Bed format information of differentially methylated RNA sites**					**Annotation**		**Comparison information**			
**Chrom**	**Tx-Start**	**Tx-End**	**Peak-ID**	**Score**	**Transcript id**	**Gene Name**	**Fold change**	**P-value**	**FDR**	**Regulation**
chr5	823503	823800	diffreps_peak_354788	4.56833901	uc010itc.3	*ZDHHC11*	94.8	<0.01	<0.01	up
chr11	69866988	69867165	diffreps_peak_79472	5.41053416	ENST00000528507	*RP11-626H12.2*	91.2	<0.01	<0.01	up
chr1	228482721	228482842	diffreps_peak_40232	4.53073455	ENST00000494839	*OBSCN*	88.6	<0.01	<0.01	up
chr5	59817681	59818280	diffreps_peak_364503	4.46045373	NR_028509	*PART1*	85	<0.01	<0.01	up
chr22	27282871	27283220	diffreps_peak_283566	3.94298158	ENST00000422915	*RP1-40G4P.1*	79.9	<0.01	<0.01	up
chr3	49823541	49823632	diffreps_peak_297612	4.53825626	ENST00000498149	*IP6K1*	76.3	<0.01	<0.01	up
chr7	143097918	143098220	diffreps_peak_443082	4.53825626	ENST00000497891	*EPHA1*	76.3	<0.01	<0.01	up
chr5	156533637	156534080	diffreps_peak_383198	3.46633137	ENST00000521665	*HAVCR2*	71.2	<0.01	<0.01	up
chr1	156426144	156426369	diffreps_peak_28036	3.55861964	ENST00000452465	*RP11-98G7.1*	71	<0.01	<0.05	up
chr9	115873689	115873957	diffreps_peak_489373	3.32546273	NR_024376	*FAM225B*	67.7	<0.01	<0.05	up
chr9	141093587	141093775	diffreps_peak_495149	3.95028224	ENST00000428088	*RP11-885N19.6*	67.5	<0.01	<0.01	up
chr6	133421201	133421323	diffreps_peak_411528	3.88579957	NR_026969	*LINC00326*	66.6	<0.01	<0.01	up
chrX	25911480	25911580	diffreps_peak_499293	3.88579957	ENST00000423914	*RP11-86A5.1*	66.6	<0.01	<0.01	up
chr17	41895286	41895420	diffreps_peak_186032	3.24273181	ENST00000589375	*MPP3*	65.9	<0.01	<0.05	up
chr1	94714968	94715048	diffreps_peak_18410	3.10318774	ENST00000413103	*RP11-148B18.1*	62.5	<0.01	<0.05	up
chr7	55742856	55743660	diffreps_peak_427429	3.64470037	ENST00000432235	*RP11-310H4.6*	62.3	<0.01	<0.05	up
chr15	54239820	54239920	diffreps_peak_150380	3.25270519	ENST00000558866	*RP11-643A5.2*	61.3	<0.01	<0.05	up
chr3	125804261	125804295	diffreps_peak_310126	3.25270519	ENST00000511301	*RP11-124N2.1*	61.3	<0.01	<0.05	up
chr1	166056421	166056660	diffreps_peak_29861	3.04738069	ENST00000366136	*RP11-375H19.2*	60.6	<0.01	<0.05	up

chr17	34242801	34243240	diffreps_peak_184150	3.047380686	ENST00000586565	*LRRC37A9P*	60.6	<0.01	<0.05	up
chr7	66022261	66022349	diffreps_peak_429008	6.34079187	ENST00000325130	*AC006001.1*	125.7	<0.01	<0.01	down
chr10	131908967	131909081	diffreps_peak_64320	7.27730939	NR_034125	*LINC00959*	117.1	<0.01	<0.01	down
chr2	196860321	196860404	diffreps_peak_255947	5.23658737	ENST00000447755	*AC104600.1*	104.7	<0.01	<0.01	down
chr12	9280378	9281000	diffreps_peak_92882	5.59841883	ENST00000540749	*RP11-436I9.5*	91.4	<0.01	<0.01	down
chr4	9159204	9159285	diffreps_peak_326053	4.80954578	ENST00000509817	*FAM86KP*	90.9	<0.01	<0.01	down
chr21	45545541	45545780	diffreps_peak_279409	4.57103674	ENST00000471490	*PWP2*	88.6	<0.01	<0.01	down
chr4	146092061	146092320	diffreps_peak_347936	4.57103674	ENST00000504331	*OTUD4*	88.6	<0.01	<0.01	down
chr1	6294464	6294560	diffreps_peak_1889	14.0000101	ENST00000489498	*ICMT*	88	<0.01	<0.01	down
chr16	29086162	29086442	diffreps_peak_166439	5.39821307	NR_003369	*RRN3P2*	87.1	<0.01	<0.01	down
chr1	28210421	28210760	diffreps_peak_6119	4.29509258	ENST00000492877	*THEMIS2*	84.3	<0.001	<0.01	down
chr17	70078262	70078556	diffreps_peak_191118	4.78368913	ENST00000602213	*SOX9-AS1*	81.3	<0.01	<0.01	down
chr16	25027298	25027820	diffreps_peak_165721	4.78368913	ENST00000569988	*RP11-266L9.1*	81.3	<0.01	<0.01	down
chr1	168761568	168762100	diffreps_peak_30248	4.78368913	NR_024160	*LINC00626*	81.3	<0.01	<0.01	down
chr19	28221681	28221920	diffreps_peak_214222	4.05042144	uc002nrw.1	*AK094188*	79.2	<0.01	<0.01	down
chr5	79818001	79818220	diffreps_peak_368503	4.05087713	ENST00000509292	*FAM151B*	79	<0.01	<0.01	down
chr8	145305114	145305360	diffreps_peak_469592	4.06947926	ENST00000527552	*MROH1*	78.5	<0.01	<0.01	down
chr19	12875361	12875731	diffreps_peak_210844	3.86416527	ENST00000592259	*HOOK2*	74.9	<0.01	<0.01	down
chr12	88427541	88427814	diffreps_peak_107634	3.8192279	ENST00000546547	*C12orf50*	73.7	<0.01	<0.01	down
chr2	202897641	202898000	diffreps_peak_257167	3.87304801	ENST00000608741	*RP11-107N15.1*	73.2	<0.01	<0.01	down
chr15	86839541	86840220	diffreps_peak_157234	4.431926698	ENST00000564487	*AGBL1-AS1*	72.6	<0.01	<0.01	down

Differentially methylated RNA sites for: Case vs Control; Fold change cut-off: 2; P-value cut-off: 0.01; FDR: the corrected p-value between two groups; Regulation: up or down in test group; Transcript id: the unique identifier describing the predicted lncRNA assigned by stringtie; Gene Name: the gene id assigned by stringtie

Supplementary Table 6 66 hub-lncRNAs overlapped between METTL3 knockdown by different shRNAs in MC-40 and HGC-27 cells

Annotations					MC-40 shMETTL3/shNC Comparison information			HGC-27 shMETTL3/ shNC Comparison information		
**Gene Name**	**Transcript id**	**tx-Start**	**tx-End**	**Peak-ID**	**Fold change**	**P-value**	**FDR**	**Fold change**	**P-value**	**FDR**
AC025165.8	ENST00000356672	58011781	58011848	diffreps_peak_102370	2.07	<0.01	<0.01	21.30	<0.05	0.41
ANKRD20A18P	ENST00000359341	15436645	15436848	diffreps_peak_275299	3.57	<0.01	<0.01	2.02	<0.05	0.26
DGKZP1	ENST00000378718	44542559	44542840	diffreps_peak_118724	3.74	<0.01	<0.01	7.43	<0.05	0.28
PSG6	ENST00000402456	43414061	43414700	diffreps_peak_217953	7.56	<0.01	<0.01	2.89	<0.01	<0.01
CICP5	ENST00000424045	224137222	224137516	diffreps_peak_39040	2.58	<0.01	<0.01	2.53	<0.01	<0.01
CICP7	ENST00000432723	330621	331120	diffreps_peak_120	2.08	<0.01	<0.01	2.41	<0.01	0.01
OFD1P17	ENST00000437639	37211381	37211760	diffreps_peak_360031	11.98	<0.01	<0.01	3.07	<0.01	0.02
CICP3	ENST00000440782	658601	659740	diffreps_peak_215	6.16	<0.01	<0.01	2.44	<0.01	<0.01
CICP4	ENST00000447359	62923621	62924120	diffreps_peak_274438	15.39	<0.01	<0.01	26.20	<0.05	0.30
LINC00319	ENST00000448049	44866521	44866720	diffreps_peak_279060	2.82	<0.01	<0.01	20.80	<0.05	0.42
AC068580.6	ENST00000449248	1783521	1783716	diffreps_peak_65797	2.88	<0.01	<0.01	21.30	<0.05	0.41
MPRIPP1	ENST00000452433	44621429	44622060	diffreps_peak_296026	2.07	<0.01	<0.01	2.76	<0.01	<0.01
TXNDC5	ENST00000460138	7889161	7889380	diffreps_peak_388886	3.26	<0.01	<0.01	5.84	<0.01	<0.01
AXIN1	ENST00000461023	340721	341040	diffreps_peak_160163	2.27	<0.01	<0.01	63.00	<0.01	0.02
APP	ENST00000464867	27270061	27270260	diffreps_peak_276338	2.10	<0.01	<0.01	3.79	<0.01	<0.01
KLHL29	ENST00000471654	23912301	23912520	diffreps_peak_227000	3.16	<0.01	<0.01	3.58	<0.05	0.20
LPCAT1	ENST00000475622	1457861	1458160	diffreps_peak_354996	8.40	<0.01	<0.01	27.20	<0.05	0.30
MAMDC4	ENST00000479475	139754161	139754440	diffreps_peak_494424	2.22	<0.01	<0.01	46.50	<0.01	0.04
TNK2	ENST00000481865	195612521	195612780	diffreps_peak_322135	2.45	<0.01	<0.01	31.50	<0.05	0.18
RXRA	ENST00000484822	137295601	137295980	diffreps_peak_493599	2.02	<0.01	<0.01	21.30	<0.05	0.41
EML3	ENST00000494448	62379701	62379853	diffreps_peak_76640	4.03	<0.01	<0.01	5.74	<0.01	0.09

TMEM110-MUSTN1	ENST00000495552	52869821	52870300	diffreps_peak_298676	13.89	<0.01	<0.01	2.28	<0.05	0.30
RMND5B	ENST00000507937	177572970	177573140	diffreps_peak_385998	2.00	<0.01	<0.01	2.98	<0.01	0.02
ST3GAL1	ENST00000519435	134582561	134582622	diffreps_peak_467677	6.12	<0.01	<0.01	2.15	<0.01	<0.01
RP11-219B4.3	ENST00000520129	86089941	86090033	diffreps_peak_459537	3.23	<0.01	<0.01	2.01	<0.01	0.04
PIDD	ENST00000527357	801481	801720	diffreps_peak_65535	2.24	<0.01	<0.01	3.74	<0.05	0.23
NADSYN1	ENST00000527538	71170720	71170860	diffreps_peak_79848	9.78	<0.01	<0.01	2.73	<0.01	<0.01
C12orf60	ENST00000527783	15058121	15058320	diffreps_peak_94165	116.50	<0.01	<0.01	21.30	<0.05	0.41
EFEMP2	ENST00000533347	65640153	65640413	diffreps_peak_78064	2.32	<0.01	<0.01	3.95	<0.05	0.22
MC1R	ENST00000539976	89978526	89978580	diffreps_peak_176803	2.84	<0.01	<0.01	7.47	<0.01	<0.01
METTL3	ENST00000545788	21978801	21979122	diffreps_peak_127854	2.20	<0.01	<0.01	2.87	<0.01	<0.01
RELT	ENST00000545886	73103961	73104360	diffreps_peak_80427	2.65	<0.01	<0.01	2.77	<0.01	0.03
RP11-424I19.2	ENST00000557903	101710754	101710880	diffreps_peak_159730	2.60	<0.01	<0.01	6.65	<0.01	0.04
RP11-505K9.3	ENST00000563242	84154694	84154700	diffreps_peak_174279	117.50	<0.01	<0.01	3.11	<0.05	0.24
AC004449.6	ENST00000564240	637104	637537	diffreps_peak_206073	2.73	<0.01	<0.01	2.78	<0.01	0.00
RP11-95P2.3	ENST00000573220	4230117	4230140	diffreps_peak_162727	5.54	<0.01	<0.01	42.70	<0.01	0.10
ZZEF1	ENST00000573606	3936341	3936560	diffreps_peak_178399	2.52	<0.01	<0.01	6.30	<0.01	0.10
TFAP4	ENST00000575320	4315761	4315980	diffreps_peak_162752	2.86	<0.01	<0.01	30.50	<0.05	0.21
MNT	ENST00000575402	2293301	2293640	diffreps_peak_177892	8.06	<0.01	<0.01	2.30	<0.01	0.16
KCTD1	ENST00000577255	24036821	24037120	diffreps_peak_198643	6.39	<0.01	<0.01	25.60	<0.05	0.33
RP11-573D15.9	ENST00000577781	186500281	186501040	diffreps_peak_320785	7.20	<0.01	<0.01	5.13	<0.01	<0.01
TP53I13	ENST00000579674	27899701	27900174	diffreps_peak_182886	3.86	<0.01	<0.01	2.66	<0.01	0.02
GRIN2C	ENST00000584176	72842898	72843400	diffreps_peak_191511	109.30	<0.01	<0.01	2.87	<0.01	0.14
PRKACA	ENST00000587533	14207182	14207540	diffreps_peak_211228	4.00	<0.01	<0.01	4.86	<0.01	0.02
TBC1D3G	ENST00000587756	34801441	34801605	diffreps_peak_184224	2.96	<0.01	<0.01	26.20	<0.05	0.30
THOP1	ENST00000589087	2807961	2808240	diffreps_peak_207272	2.17	<0.01	<0.01	2.75	<0.05	0.30
FGF22	ENST00000591390	640001	640900	diffreps_peak_206081	229.60	<0.01	<0.01	8.09	<0.01	<0.01

PNPLA6	ENST00000594754	7599635	7600260	diffreps_peak_208990	2.01	<0.01	<0.01	2.19	<0.01	<0.01
JOSD2	ENST00000595718	51013396	51013540	diffreps_peak_221082	2.85	<0.01	<0.01	61.00	<0.01	0.02
GS1-393G12.14	ENST00000607491	145635021	145635360	diffreps_peak_469871	5.82	<0.01	<0.01	2.70	<0.01	0.17
MYH16	NR_002147	98885514	98885760	diffreps_peak_434907	5.00	<0.01	<0.01	3.46	<0.01	0.05
SNHG7	NR_003672	139622621	139622636	diffreps_peak_494315	2.60	<0.01	<0.01	3.49	<0.01	0.11
PLEKHM1P	NR_024386	62781001	62781240	diffreps_peak_190180	2.80	<0.01	<0.01	8.40	<0.01	<0.01
KTN1-AS1	NR_027123	56043381	56043980	diffreps_peak_134695	3.94	<0.01	<0.01	2.71	<0.01	0.01
TRAF3IP2-AS1	NR_034108	111920341	111920560	diffreps_peak_407742	2.06	<0.01	<0.01	7.52	<0.05	0.28
PTOV1-AS1	NR_040037	50347572	50347880	diffreps_peak_220846	8.27	<0.01	<0.01	6.55	<0.001	0.03
RNASEH2B-AS1	NR_046552	51484618	51484848	diffreps_peak_120029	103.80	<0.01	<0.01	3.45	<0.05	0.22
TONSL-AS1	NR_109770	145664858	145665040	diffreps_peak_469913	2.18	<0.01	<0.01	3.94	<0.05	0.18
LOC101928034	NR_125947	153523718	153524080	diffreps_peak_27145	4.61	<0.01	<0.01	2.91	<0.05	0.40
AK128439	uc002ezo.1	70723141	70723460	diffreps_peak_172397	3.25	<0.01	<0.01	2.85	<0.01	<0.01
BC037357	uc002goo.3	15332289	15332340	diffreps_peak_180377	2.77	<0.01	<0.01	3.31	<0.01	0.15
AX747521	uc002jrt.3	74493021	74493240	diffreps_peak_192120	3.74	<0.01	<0.01	26.20	<0.05	0.30
RP11-392P7.6	ENST00000538231	13101128	13101420	diffreps_peak_93904	16.39	<0.01	<0.01	47.00	<0.01	0.04
DNM1P47	ENST00000561463	102303281	102303660	diffreps_peak_159935	2.16	<0.01	<0.01	35.80	<0.01	0.15
SLC27A5	ENST00000593745	59021881	59022280	diffreps_peak_223900	12.28	<0.01	<0.01	21.30	<0.05	0.41
RP4-758J18.13	ENST00000607307	1344521	1344820	diffreps_peak_760	2.97	<0.01	<0.01	3.50	<0.05	0.26

Differentially methylated RNA sites for Case vs Control； Fold change cut-off: 2； P-value cut-off: 0.01； FDR: the corrected p-value between two groups； Regulation: up or down in test group； Transcript id: the unique identifier describing the predicted lncRNA assigned by stringtie；Gene Name: the gene id assigned by stringtie

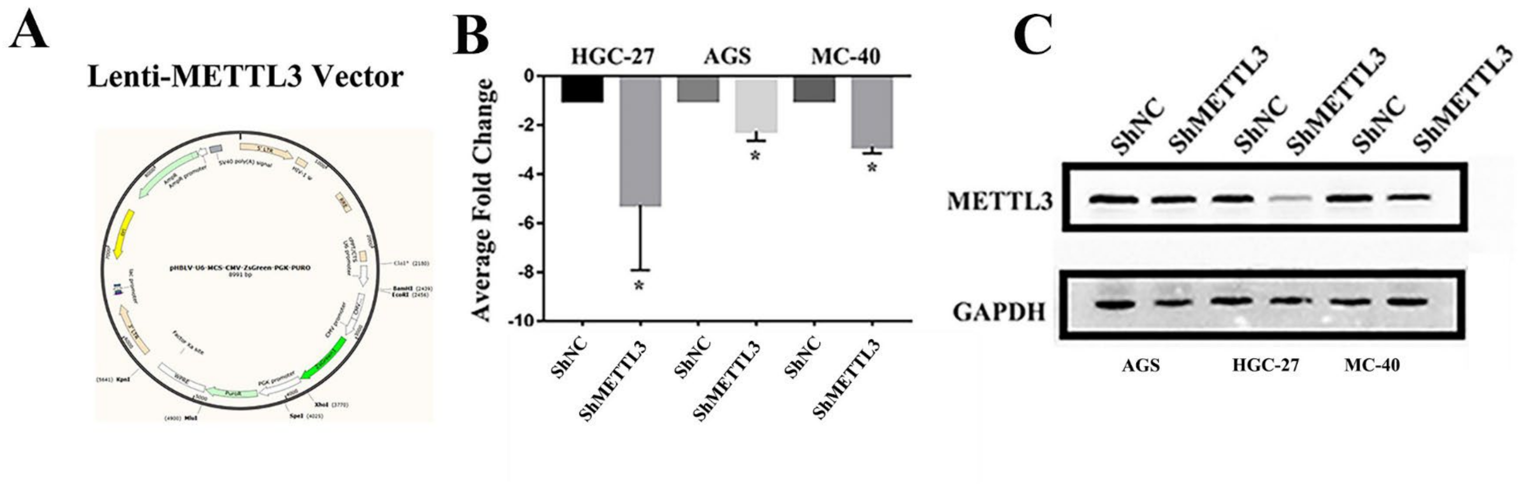

Supplementary Figure 1 Knock-down of METTL3 in MC and GC cells. (A) Lentiviral vectors stably knocking down METTL3 were infected with MC and GC cells. (B) qRT-PCR detected the transfected efficiency, *$P<0.05$. (C) Western Blot analysis confirmed the transfected efficiency.

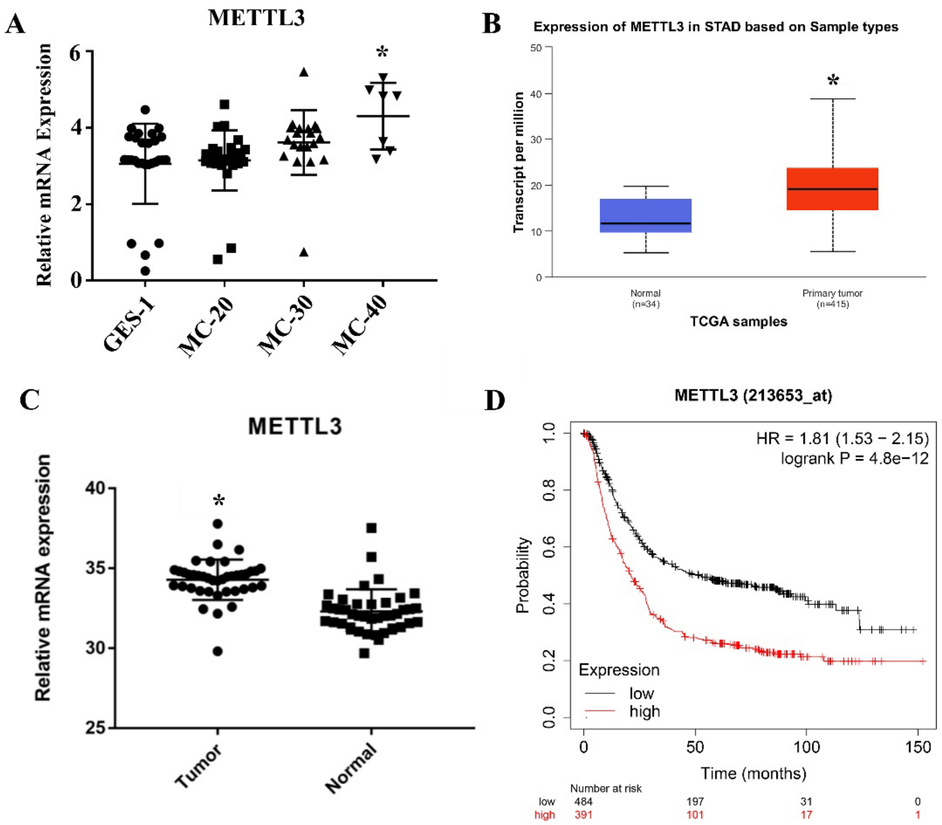

Supplementary Figure 2 METTL3 expression in GC cells and patients. A. METTL3 mRNA expression levels in MC cells, *P<0.05; B. METTL3 mRNA expression levels in TCGA STAD cohort, *P<0.05; C. METTL3 mRNA expression levels in GC tissue samples, *P<0.05; D. The Kaplan–Meier estimates survival time in two groups of patients from the GEO GC cohort by different METTL3 expression levels.
